# Dataset of concurrent EEG, ECG, and behavior with multiple doses of transcranial electrical stimulation

**DOI:** 10.1038/s41597-021-01046-y

**Published:** 2021-10-27

**Authors:** Nigel Gebodh, Zeinab Esmaeilpour, Abhishek Datta, Marom Bikson

**Affiliations:** 1 The Department of Biomedical Engineering, The City College of New York, The City University of New York, New York, USA; 2 Soterix Medical Inc., New York, USA

**Keywords:** Attention, Biophysical models, Predictive markers, Neurophysiology, Brain-machine interface

## Abstract

We present a dataset combining human-participant high-density electroencephalography (EEG) with physiological and continuous behavioral metrics during transcranial electrical stimulation (tES). Data include within participant application of nine High-Definition tES (HD-tES) types, targeting three cortical regions (frontal, motor, parietal) with three stimulation waveforms (DC, 5 Hz, 30 Hz); more than 783 total stimulation trials over 62 sessions with EEG, physiological (ECG, EOG), and continuous behavioral vigilance/alertness metrics. Experiment 1 and 2 consisted of participants performing a continuous vigilance/alertness task over three 70-minute and two 70.5-minute sessions, respectively. Demographic data were collected, as well as self-reported wellness questionnaires before and after each session. Participants received all 9 stimulation types in Experiment 1, with each session including three stimulation types, with 4 trials per type. Participants received two stimulation types in Experiment 2, with 20 trials of a given stimulation type per session. Within-participant reliability was tested by repeating select sessions. This unique dataset supports a range of hypothesis testing including interactions of tDCS/tACS location and frequency, brain-state, physiology, fatigue, and cognitive performance.

## Abstract (summary)

**Table:** 

Measurement(s)	brain measurement • physiological measurement • behavioral measurement • Heart Rate • Attention Concentration • Pittsburgh Sleep Quality Index • Hours of Actual Sleep at Night • Sleepiness • Decreased vigilance • Mood • Pain or Discomfort Today • Anxiety • Overall Energy Level • Continuous vigilance and attention
Technology Type(s)	electroencephalography • electrocardiography • Behavioral, Psychological or Informational Intervention • Pittsburgh Sleep Quality Index • Numeric Rating Scale • Karolinska Sleepiness Scale • Numeric Pain Scale • Compensatory Tracking Task
Factor Type(s)	cortical region • stimulation waveform • sex • age • stimulation location • weight • height
Sample Characteristic - Organism	Homo sapiens

Machine-accessible metadata file describing the reported data: 10.6084/m9.figshare.16558119

## Background & Summary

Transcranial electrical stimulation (tES), including its variants: transcranial Direct Current Stimulation (tDCS) and transcranial Alternating Current Stimulation (tACS), allows for testing of causal relationship between brain function and cognition^1–3^. In recent years, hundreds of tES trials have broadly impacted fields of cognitive neuroscience and behavioral performance^4,5^, alongside intensive testing as clinical treatments^6–8^. tES efficacy is rationalized to depend on waveform (AC frequency or DC) and targeted brain region. The use of High-Definition tES (HD-tES) allows for localized targeting of cortical regions^9^, which can be combined with variations in stimulation waveform^10–12^.

The combination of tES with brain imaging techniques^13–17^, notably EEG, allows for verification of neural target engagement^7,8,18^, optimization of interventions^10,19^, and powerful analysis of brain structure and functional relationships^3^. Analysis of online tES-EEG experiments are not without technical challenges^20–23^.

In the field of brain stimulation or neuromodulation, the development of open-source tools for modeling pipelines^24–28^ and signal processing^29^ have far surpassed the availability of data. With rare exceptions^30–32^, available brain stimulation datasets are centered on validating current flow^33,34^, providing anatomical templates^35,36^, or are derived from animal models^37^. The lack of raw data sharing and availability in the neuromodulation field can lead to decreased transparency when it comes to examining tES-EEG relationships, verifying analyses or analysis-dependent^38^ relationships in tES-EEG outcomes, as well as a decrease in the democratization of algorithmic development, especially when it comes to enhancing algorithms geared toward closed-loop tES.

The effects of tES on attention and vigilance^17,39–41^ have been extensively studied, and are of universal interest with regard to tES, since they impact applications such as accelerated learning^42^, neurorehabilitation^6,43^ and neuro-psychiatric treatment^44–46^. An openly available dataset with continuous metrics of vigilance/attention, over an extended period of time, paired with concurrent EEG and physiologic metrics, during varied types of tES would present as a significant contribution to the field of neuromodulation.

To test how vigilant/attentional states are acutely altered by specific tES types, over an extended period (~70 mins) of behavioral task performance, and how temporal sensitivity to stimulation is modulated by or reflected in brain/physiological state: our multimodal dataset combines human-participant, concurrent multichannel tES; multichannel EEG (sampled at 2 kHz), bipolar ECG and EOG monitoring; and continuous (in time and score) vigilance/alertness behavioral metrics. We apply 9 different electrical stimulation montages (3 stimulation locations and 3 frequencies), with each stimulation type applied 4-20 times per experimental session, in a repeated-measures crossover design – with additional repetitions for within-participant reliability. A verified^47–53^ compensatory tracking task (CTT) allowed for the assessment of vigilance/attention on the scale of milliseconds, facilitating its acquisition concurrency with dynamic EEG, ECG, and EOG. These multimodal assessments allow for the testing of 1) how variations in brain state (e.g. baseline vigilance) or physiology impact sensitivity to tES; and 2) how tES changes brain state or physiology (e.g. autonomic function as indicated by heart rate variability) along with changes in performance.

Our approach varies from typical tES interventions in applying stimulation since we utilize short stimulation (30 sec) epochs, in contrast to tens of minutes. Although it has been observed that tens of minutes of stimulation enhances the detection of stimulation related after-effects^54–57^ such effects come on the heels of and are resultant from an accumulation of online (immediate) effects^58–61^, which are currently challenging to directly observe. Modulatory EEG effects are typically rapid, on the order of milliseconds^10,18,20,23,62^, as are cellular responses in animal models^63–67^. Similarly, ongoing changes in brain state, physiology, and performance (e.g. fatigue/vigilance shift) can be rapid^68–72^ too. Closed-loop stimulation approaches require the use of transient biomarkers. Ultimately, our selection of short stimulation epochs was driven by: 1) the aforementioned mechanistic factors; 2) the value of increasing statistical power (repetitions) and signal to noise ratio (e.g. minimizing drift over time); as well as supporting algorithm development for 3) data cleaning (e.g. online EEG artifact correction^21,22^) and 4) closed-loop (e.g. machine learning) optimization - noting any sufficiently large open-loop experiments can be analyzed as closed-loop^73–75^ interventions. The complete dataset and assessments are provided without restriction under the Creative Commons with Attribution 4.0 license and is detailed in this manuscript. We hope the availability of this dataset will alleviate barriers in the field of tES and allow for the exploration and testing of multiple hypotheses associated with the design of optimized stimulation interventions.

## Methods

### Participants

**Fig. 1:**
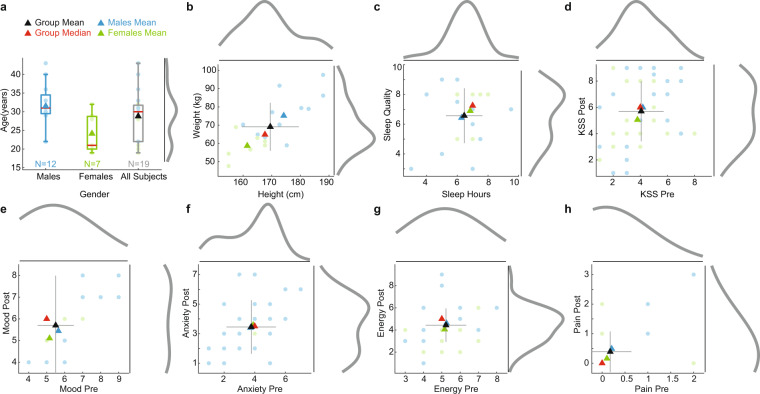
Demographic and questionnaire summary. (**a**) Included participants’ ages ranged from 19-43 years old with a mean age of 28.79 years. (**b**) Included participants’ mean height and weight were 169.50 cm and 69.11 kg, respectively. (**c**) The mean sleep hours and sleep quality ratings were 6.45 hrs and 6.57 (5-Normal Quality), respectively. (**d**) The mean sleepiness ratings (KSS) before (*KSS Pre*) and after (*KSS Post*) each session was 4.01 and 5.70, respectively. (**e**) The mean mood ratings before (*Mood Pre*) and after (*Mood Post*) each session was 5.50 and 5.70 (5-Usual self), respectively. (**f**) The mean anxiety ratings before (*Anxiety Pre*) and after (*Anxiety Post*) each session were 3.77 and 3.45 (5-Usual self), respectively. (**g**) The mean energy ratings before (*Energy Pre*) and after (*Energy Post*) each session were 3.77 and 3.45 (5-Usual self), respectively. (**h**) The mean pain ratings before (*Pain Pre*) and after (*Pain Post*) each session was 0.18 and 0.39 (0-No pain, 10-Worst possible pain), respectively. For panels (**c–h**) data are illustrated for the aggregate of all sessions (including participants’ repeated sessions) and for (**b–h**) gray lines extending from each group mean indicate one standard deviation.

A total of twenty neurologically typical individuals (7 females, 13 males) between the ages of 19 − 43 (median age: 30; mean age: 29.10 ± 6.75) were recruited from the New York metropolitan area. Before enrolment, participants underwent a tES eligibility screening procedure where they were asked questions regarding their medical history, psychiatric history, and prior drug use (non/illicit) including history of seizures, depression, surgeries, pain, ear trauma, heart conditions, skin allergies, implantable medical devices (non/metallic), alcohol dependency, brain lesions, loss of consciousness, sleep disorders, and fainting at the sight of blood. Participants who met the eligibility criteria were then enrolled (see Fig. 1a,b). After passing the eligibility screening, informed consent was obtained for each participant. Experimental procedures were reviewed and approved by the Western Institutional Review Board and all procedures were conducted in accordance with the ethical guidelines set forth by the Declaration of Helsinki in 1964 and its later amendments. All participants were financially compensated for their participation.

Prior to each experimental session participants were asked to maintain their normal sleep-wake cycle, refrain from consuming alcohol or overly caffeinated foods/ beverages at least 4 h before their session, and refrain from eating any heavy meals at least 3 h before their visit. In order to facilitate expeditious EEG set-up, participants were also asked to refrain from wearing any heavy make-up or facial moisturizer, and refrain from wearing any hair products or scalp treatments^76^.

One participant was excluded from the experiments (participant 17) due to inability to follow instructions in performing the experiment’s behavioral task (see Table 1). Four participants (participant number: 12, 15, 21, 22) were invited back to repeat both their experimental sessions for Experiment 2 (see *Experimental Overview*). Upon returning to repeat Experiment 2, returning participants were assigned a new participant number based on the incremental sequence of participant number assignment (i.e. 1^st^ participant enrolled assigned to participant number 01, 2^nd^ participant enrolled assigned to participant number 02, etc.). Participant 12 returned once and was assigned an updated participant number: 19; participant 15 returned once and was assigned an updated participant number: 18; participant 21 returned twice and was assigned updated participant numbers: 25 and 26; and participant 22 returned twice and was assigned updated participant numbers: 23 and 24 (see Table 2).

**Table 1:** Summary of all participant sessions for Experiment 1 including stimulation condition and stimulation intensity.

Sub#	Session	File Num	F0	M0	P0	F5	M5	P5	F30	M30	P30	Stim Type			Stim Amplitude (mA)		
												Block 1	Block 2	Block 3	Block 1	Block 2	Block 3
**01**	01	0101	1						7	8		M30	F30	F0^♦^	1	1	1
	02	0102	1						7	8		M30	F30	F0^♦^	0.5	0.5	0.5
	03	0103			3			6			9	P30	P0	P5	0.5	0.5	0.5
	04	0104		2		4	5					F5	M5	M0	0.5	0.5	0.5
	05	0105		2		4	5					F5	M5	M0	1	1	1
	06	0106			3			6			9	P30	P0	P5	1	1	1
**02**	01	0201				4			7		9	F30^♦^	F5	P30	0.5	0.5	0.5
	02	0202			3		5			8		P0	M5	M30	0.5	0.5	0.5
**03**	01	0301		2	3				7			M0	P0	F30	0.5	0.5	0.5
	02	0302				4		6			9	F5	P5	P30	0.5	0.5	0.5
	03	0303	1				5			8		M5	F0	M30	0.5	0.5	0.5
**04**	01	0401				4				8	9	P30	M30	F5	0.5	0.5	0.5
	02	0402	1	2	3							P0	F0	M0	1	1	1
	03	0403					5	6	7			F30	M5	P5	1	1	1
**05**	01	0501			3		5	6				M5	P0	P5	0.5	1	1
	04	0504		2						8	9	P30	M30	M0	1	1	1
	05	0505	1			4			7			F30	F0	F5	1	1	0.5
**06**	01	0601	1			4		6				F0	F5	P5	1	0.5	1
	02	0602							7	8	9	F30	M30	P30	0.5	1	1
	03	0603		2	3		5					P0	M5	M0	1	1	1
**07**	01	0701				4	5	6				P5	F5	M5	0.5	0.5	0.5
	02	0702	1	2	3							M0	P0	F0	0.5	0.5	0.5
	03	0703							7	8	9	F30	P30	M30	0.5	0.5	0.5
**08**	01	0801				4	5			8		F5	M30	M5	1	1	1
	02	0802	1		3						9	P30	F0	P0	1	1	1
	03	0803		2				6	7			P5	F30	M0	1	1	1
**09**	01	0901	1			4					9	P30	F5	F0	0.5	0.5	1
	02	0902			3		5		7			M5	F30	P0	0.5	1	1
	03	0903		2				6		8		P5	M0	M30	1	1	1
**10**	01	1001		2	3		5					M0	P0	M5	1	1	1
	02	1002	1						7		9	F0	P30	F30	1	1	1
	03	1003				4		6		8		F5	M30	P5	1	1	1

Columns indicate participant number (*Sub #*), each participant’s session number (*Session*), the corresponding file number for each session (*File Num*), a detailed breakdown of stimulation conditions that were applied for each session across stimulation blocks (*Stim Type*), and the corresponding stimulation amplitude (*Stim Amplitude*) that was applied for each stimulation condition. Blocks with trials that encountered technical errors are indicated with black diamonds (♦) under the *Stim Type* section.

**Table 2:** Summary of all participant sessions for Experiment 2 including stimulation condition and stimulation intensity.

Sub#	Session	File Num	F30	M30	Stim Type	Stim Amplitude (mA)
**11**	01	1101	7		F30	1
	02	1102		8	M30	1
**12** ^**■**^	01	1201		8	M30	1
	02	1202	7		F30	1
**13**	01	1301	7		F30	1
	02	1302		8	M30	1
**14**	01	1401		8	M30	1
	02	1402	7		F30	1
**15**^**▲**^	01	1501	7		F30	1
	02	1502		8	M30	1
**16**	01	1601		8	M30	1
	02	1602	7		F30	1
**17**	01	1701	7		F30	1
**18**^**▲**^	01	1801		8	M30	1
	02	1802	7		F30	1
**19** ^**■**^	01	1901	7		F30	1
	02	1902		8	M30	1
**20**	01	2001	7		F30	1
	02	2002		8	M30	1
**21**^**♦**^	01	2101	7		F30	1
	02	2102		8	M30	1
**22**^**∙**^	01	2201		8	M30	1
	02	2202	7		F30	1
**23**^**∙**^	01	2301	7		F30	1
	02	2302		8	M30	1
**24**^**∙**^	01	2401		8	M30	1
	02	2402	7		F30	1
**25**^**♦**^	01	2501		8	M30	1
	02	2502	7		F30	1
**26**^**♦**^	01	2601	7		F30	1
	02	2602		8	M30	1

Columns indicate participant number (*Sub #*), each participant’s session number (*Session*), the corresponding file number for each session (*File Num*), a detailed breakdown of stimulation conditions that were applied for each session across stimulation trials (*Stim Type*), and the corresponding stimulation amplitude (*Stim Amplitude*) that was applied for each stimulation condition. Matching symbols next to participants’ numbers indicate the same individual who returned for additional sessions and was assigned a new subject number. Participant 12 retuned once and was assigned a new number: 19 (**■**). Participant 15 returned and was assigned a new number: 18 (**▲**). Participant 21 returned twice and was assigned new numbers: 25 and 26 (**♦**). Participant 22 returned twice and was assigned new numbers: 23 and 24 (**∙**).

### Questionnaires

Upon enrollment participants completed a demographic questionnaire to assess their age, gender, height, weight, years of education, handedness, English proficiency, typical exercise time each week, history of electrical stimulation, and sleep quality (quantified with the Pittsburgh Sleep Quality Index; PSQI^77^). These questionnaires were only administered once upon participant’s enrollment on their initial session (hence empty cells in *GX_Subject Info & Behavioral Data.xlsx* for participants who repeated Experiment 2; see *Demographics, PSQI, and Pre Post Questionnaires*).

At the beginning of each session participants completed a prequestionnaire consisting of questions regarding their activities in the past 24 h (see *GX_Demo_PreQuest_Scales.pdf*, page 2), in addition, they also completed assessment scales immediately before and after each session (Fig. 1c–h). These scales included assessments of sleepiness (Karolinska Sleepiness Scale; a 9-point scale ranging from 1-Extremely alert to 9-Very sleepy, great effort to keep awake, fighting sleep), discomfort scale (ranging from 1-No discomfort to 9-Extreme discomfort), pain (numeric pain scale; ranging from 0-No pain to 10-Worst possible pain), mood (ranging 1-Saddest to 9-Happiest), anxiety (ranging 1-Most relaxed to 9-Most tense), and energy level (ranging 1-Most tired to 9-Most energetic). Wellness scales were modeled after the standardized English version of the Karolinka Sleepiness Scale (KSS)^78–80^, which range from 1 to 9; and the pain scale utilized was modeled after standard Numeric Rating Scales (NRS) or Numeric Pain Scales (NPS) that conventionally range from 0 to 10^81,82^.

### Behavioral task

**Fig. 2:**
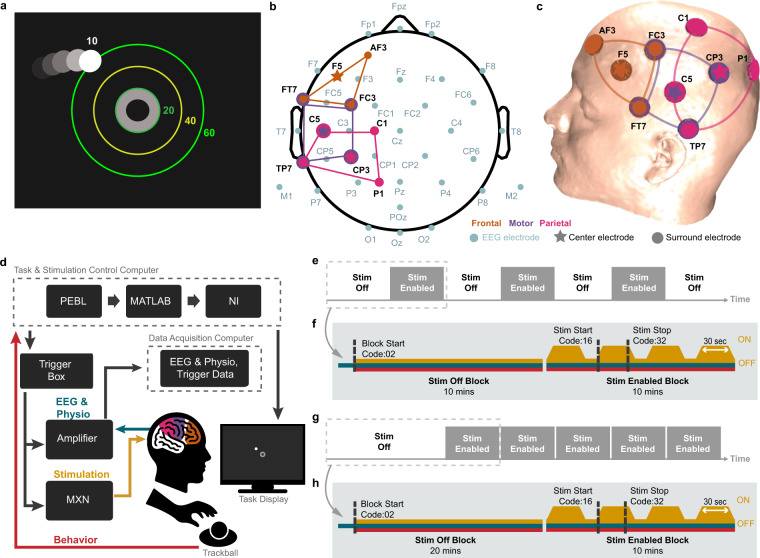
Behavioral task, EEG and stimulation montage, hardware setup, and experimental overview. (**a**) The CTT behavioral task where the objective was to maintain the moving circle (~10 pixels) near the center of the middle annulus (~20 pixels) over the course of the experiment (70 mins). The ball was endowed with inherent motion, and oscillatory and dampening forces. Note, only the white ball and gray annulus were visible to participants, in the panel additional rings and distances are for illustrative purposes. (**b**) EEG locations (light blue) interleaved with stimulation sites for frontal (orange), motor (purple), and parietal (magenta) stimulation. For each stimulation montage, center electrodes are indicted by a star and surrounding electrodes by circles. Note, by design some electrode locations may serve as a center electrode for one montage and outer electrodes for another montage. (**c**) MRI-derived 3D head model with the stimulation locations placed on the scalp to visualize electrode placement. (**d**) Data acquisition and stimulation hardware setup in relation to participants’ input and output. (**e**) Experiment 1 and (**g**) Experiment 2 block design as programed to be executed within the hardware and software setup in panel (**d**). Experiments were divided into *stimulation off* blocks (*Stim Off*) without tES and *stimulation enabled* blocks (*Stim Enabled*) within which the selected tES stimulation condition was applied 4 times. (**f**) Experiment 1 and (**h**) Experiment 2 detailed block design with corresponding EEG triggers (dashed vertical line) and trigger codes. Note that the block design is not drawn to scale. Simultaneous *EEG and Physio* (EEG, ECG, and EOG; teal); and *Behavior* (CTT, red) were acquired with concurrent *Stimulation* (gold). Note, *EEG and Physio* acquisition started several seconds before any trigger delivery; and (**e,f**) are designed to illustrate the experimental design as programmed to be executed.

Participants were seated in a dimly lit (~10 lux) room and some were offered foam in-ear ear plugs to aid in sound attenuation. Participants were seated ~57 cm in front of a 17-inch LCD monitor (Model: E173FPb; Dell Corp., Texas, USA; set at a 60 Hz refresh rate) and were asked to continuously perform a Compensatory Tracking Task (CTT) over the course of each session. On-screen task instructions presented to participants stated: *Your goal in this task is to keep the ball near the target using your mouse or pointing device. The ball moves on its own, but is impacted by how you move the mouse. If possible, keep the ball in the center of the ring, or as close as possible to the inner circle as you can*. The goal of the 2-dimensional task was to keep a moving circle constantly constrained near an annulus at the center of the screen (Fig. 2a). The circle was endowed with several kinematic properties including oscillatory and dampening forces^47^. The CTT was sourced and administered through the open source PEBL software version 2.1, an experimental psychology task program built upon C++^83^. Participants provided task input through their dominant hand via a trackball (Logitech TrackMan Marble Mouse; Model: 904360-0403; Logitech International S.A., Lausanne, Switzerland) and were left uninterrupted throughout the duration of the task (even in cases where participants were in visible hypnagogic states; consistent with prior CTT designs). Before the start of each session participants performed a short practice session that lasted between 1-3 mins. The duration of the task was set to 70 mins for both Experiment 1 and 2 (extended for an additional 0.5 mins). The CTT ran continuously and uninterrupted over the duration of both experiments and participants were blinded to each experiment’s block design and stimulation type.

### EEG data acquisition

EEG data were acquired using a wired Waveguard cap containing 32 Ag/AgCl recording channels (ANT Neuro, Hengelo, The Netherlands) and 29 interleaved plastic HD-holders (Soterix Medical Inc., New York, USA). The HD-holders were used to house the stimulation materials including conductive gel and stimulation electrodes (see *HD-tES*). For both stimulation and recording, conductive gel (SignaGel, Parker Laboratories Inc., New Jersey, USA) was placed between electrodes and the scalp using blunt tip (15-gauge; Cortech Solutions Inc., North Carolina, USA) syringes.

EEG recording electrodes were located at standard locations following the 10/10 international placement system (Fig. 2b). Signals were sampled at 2 kHz, referenced relative to CPz, online grounded at AFz, and amplified with an eego sport amplifier (ANT Neuro, Hengelo, The Netherlands). The amplifier bandwidth was set between 0-520 Hz and the acquisition voltages range was set to 1 V peak-to-peak. Prior to each recording, scalp impedances were monitored to ensure impedance levels were below 20 kΩ. To time-lock the CTT with the concurrent EEG a trigger was sent to the EEG amplifier at the start of the CTT (see *Data Records*). Following data acquisition, data were exported to a *.cnt* file.

### Physiological monitoring

Cardiac activity was acquired with a lead I electrocardiogram (ECG) configuration and ocular motor activity with horizontal electrooculogram (EOG) configuration. Both were acquired with bipolar snap electrodes, concurrently with EEG and HD-tES. For lead I ECG, bipolar snap electrodes were placed on participants’ chest approximately 5 cm below the center of their left (anode) and right (cathode) clavicle bone. A ground electrode was placed on participants’ left hip, in close proximity to participants’ iliac crest. For horizontal EOG, electrodes were placed at the outer canthus of the participants’ left (anode) and right (cathode) eye. Prior to all peripheral electrode placement, the application sites on the skin were gently swabbed with alcohol pads (BD Alcohol Swabs, Becton Dickinson, New Jersey, USA) to increase electrode adhesion and contact quality.

### HD-tES

HD-tES was applied for 30-sec epochs per trial with an additional 5-sec ramp-up/down period, at 3 different cephalic locations with 3 different stimulation waveforms. The 9 stimulation doses (combination of stimulation location: 3; frequency: 3; duration: 1) were characterized by the cephalic area of stimulation: frontal, motor, and parietal; as well as the frequency of the stimulation current applied: 0 (DC), 5, and 30 Hz. Each combination of stimulation location and frequency was denoted with the first letter of the stimulation location in combination with the Arabic numeral denoting the frequency. As such, the nine possible dose combinations were as follows: frontal DC (F0), frontal 5 Hz (F5), frontal 30 Hz (F30), motor DC (M0), motor 5 Hz (M5), motor 30 Hz (M30), parietal DC (P0), parietal 5 Hz (P5), parietal 30 Hz (P30).

A total of 9 Ag/AgCl sintered ring stimulation electrodes (Soterix Medical Inc., New York, USA) were placed at standard EEG 10/10 locations in manner forming three possible Nx1 HD-tES^84^; where N = 3, 4, 4 for frontal, motor, and parietal, respectively. For each montage, N electrodes were selected for the outer (surround) ring electrode, and one electrode was selected as the center electrode. Note, some electrode locations were shared across montages with varied “ring” or “center” assignments. In this way, a single 9 electrode position HD-tES set-up was prepared for all experiments. For frontal stimulation, outer or surround electrodes (N = 3) were placed at AF3, FT7, FC3 and the return or center electrode was placed at F5 (Fig. 2b,c). For motor stimulation outer or surround electrodes (N = 4) were placed at FT7, FC3, CP3, TP7, and the return or center electrode was placed at C5 (Fig. 2b,c). For parietal stimulation outer or surround electrodes (N = 4) were placed at C5, C1, P1, TP7 and the return or center electrode was placed at CP3 (Fig. 2b,c). To visualize stimulation electrode placement in realistic 3D space, an MRI-derived head model was used together with the ROAST toolbox^27,85^ in MATLAB (2018b and 2019b; MathWorks, Massachusetts, USA). Each montage shared stimulation sites and no two stimulation montages were applied at the same time.

In terms of stimulation waveforms, a monophasic DC (0 Hz) or biphasic sinusoidal waveform (5 or 30 Hz) was applied. For DC, for each cephalic location (frontal, motor, parietal) the respective center electrode was used as the cathode with the surround electrodes as anodes. For biphasic sinusoidal stimulation all electrodes switched between being an anode and cathode at a rate set by the frequency of stimulation (i.e. 5 Hz or 30 Hz).

Using conventional procedures^86^, stimulation electrodes were placed in plastic HD-holders (Soterix Medical Inc., New York, USA), which were embedded in the EEG cap (interleaved with EEG electrode positions). To prepare for electrode placement, participants’ hair was parted with a blunt Q-tip or blunt tip syringe at each stimulation location through the annulus of the plastic HD-holders, then the HD-holders were slowly backfilled with ~15 mL of HD gel (Soterix Medical Inc., New York, USA). The stimulation electrodes were then placed in the HD-holders and were fully submerged in electrolyte gel, then the plastic holder was covered with the associated plastic covering to secure the electrode in place.

Stimulation was delivered using the MxN 9-channel high-definition transcranial electrical stimulation stimulator (Soterix Medical Inc., New York, USA). The multichannel stimulation device supplied a current controlled current source where the current amplitude of each individual stimulation channel could be adjusted. Stimulation channel impedances were measured relative to F5, prior to the commencement of stimulation. During each session, the stimulator was triggered using a pulse generated in MATLAB and relayed to a National Instruments (NI) -DAQmx device (National Instruments Inc., Texas, USA). A corresponding trigger was sent to the EEG amplifier to time-lock stimulation and EEG (Fig. 2d). For details on stimulation settings for each stimulation dose see Table 3.

**Table 3:** Stimulation device (MxN) settings used for each stimulation dose.

		Frontal		Motor		Parietal	
**Waveform**		Sinusoid*		Sinusoid*		Sinusoid*	
**Polarity**		Biphasic		Biphasic		Biphasic	
**Frequency (Hz)**		0 | 5 | 30		0 | 5 | 30		0 | 5 | 30	
**Ramp Duration (secs)**		5		5		5	
**Center Electrode**		F5		C5		CP3	
**Channel Settings For Total Current at 1 and 0.5 mA**	***Channel***	***Intensity (1 mA)***	***Intensity (0.5 mA)***	***Intensity (1 mA)***	***Intensity (0.5 mA)***	***Intensity (1 mA)***	***Intensity (0.5 mA)***
	**1 (AF3)**	−0.33	−0.17	0	0	0	0
	**2 (FT7)**	−0.33	−0.17	−0.25	−0.13	0	0
	**3 (FC3)**	−0.33	−0.17	−0.25	−0.13	0	0
	**4 (C5)**	0	0	+ 1.00	+ 0.52	−0.25	−0.13
	**5 (TP7)**	0	0	−0.25	−0.13	−0.25	−0.13
	**6 (C1)**	0	0	0	0	−0.25	−0.13
	**7 (CP3)**	0	0	−0.25	−0.13	+ 1.00	+ 0.52
	**8 (P1)**	0	0	0	0	−0.25	−0.13
	**9 (F5)**^**$**^	+ 0.99	+ 0.51	0	0	0	0
	**Net Amplitude Delivered (mA)**	**0.99**	**0.51**	**1**	**0.52**	**1**	**0.52**

Each row indicates an aspect of programing the MxN stimulator. For setting stimulation frequency, either 0, 5 or 30 was selected based on the intended frequency of stimulation. The ramp duration setting was identical for stimulation ramp-up as well as stimulation ramp-down. *The MxN accepts 0 Hz as a monophasic DC. ^$^Channel 9 was automatically set to the residual sum of the programable channels (channels 1-8).

Prior to the start of each session participants underwent a pre stimulation test to determine their tolerance and comfort with each stimulation montage. Stimulation was delivered for approximately 20 secs (5-sec ramp-up/down) at an initial net current of 1 mA (peak-to-peak) and 0.5 mA (peak-to-peak) for Experiment 1 and 2, respectively. Participants were then asked to rate the level of pain/discomfort on a numeric pain scale (0-No pain/discomfort to 10-Worst pain you’ve ever felt). For Experiment 1, if participants rated a 5 or above, the stimulation intensity was halved (0.5 mA minimum amplitude); whereas for Experiment 2 if participants rated below 5, the stimulation intensity was increased to 1 mA. In both experiments the adjusted stimulation intensity was 0.5 mA at minimum and was retested to ensure that it was well tolerated. All participants in Experiment 2 received 1 mA of stimulation (see Table 2) whereas stimulation intensities for Experiment 1 were either 0.5 or 1 mA, based on pre stimulation testing (see Table 1).

### Experimental overview

In Experiment 1, each participant engaged in three 70-min sessions whereas in Experiment 2 participants engaged in two 70.5-min sessions. Prior to and after each session (for both Experiment 1 and 2), participants completed a series of pre- and post-questionnaires (see *GX_Demo_PreQuest_Scales.pdf*). For each session participants performed the CTT continuously over the course of 70 mins (or 70.5 mins) while EEG and physiology (ECG, EOG) were recorded. For Experiment 1, nine different stimulation conditions were used (F0, F5, F30, M0, M5, M30, P0, P5, P30), whereas for Experiment 2, two different stimulation conditions were used (F30, M30).

For Experiment 1, each session (of the 3 sessions per participant) consisted of three stimulation enabled blocks (10 mins each) interleaved with four stimulation off blocks (10 mins each). For each session, three of the nine total stimulation conditions were pseudo-randomly assigned to stimulation enabled blocks - to ensure participants received each of the nine stimulation conditions across the nine total stimulation enabled blocks in Experiment 1. During each stimulation enabled block the assigned stimulation condition was administered as 4 consecutive stimulation trials (see Fig. 2e,f). Each stimulation trial consisted of a 5-sec ramp-up period, 30-sec stimulation period, and a 5-sec ramp down period.

For Experiment 2, each of the two sessions (one session for F30 and one session for M30) consisted of one stimulation off blocks (20 mins), which was followed by five consecutive stimulation enabled blocks (10 mins each; Fig. 2g,h). For each session, only one of the two stimulation conditions (F30 or M30) were pseudo-randomly assigned to be administered during the five stimulation enabled blocks. The assigned stimulation condition was administered as 4 consecutive trials during each stimulation enabled blocks. Thus, a total of 20 trials of stimulation (30 secs each plus 5-sec ramp up and down) were delivered for each session in Experiment 2.

**Fig. 3:**
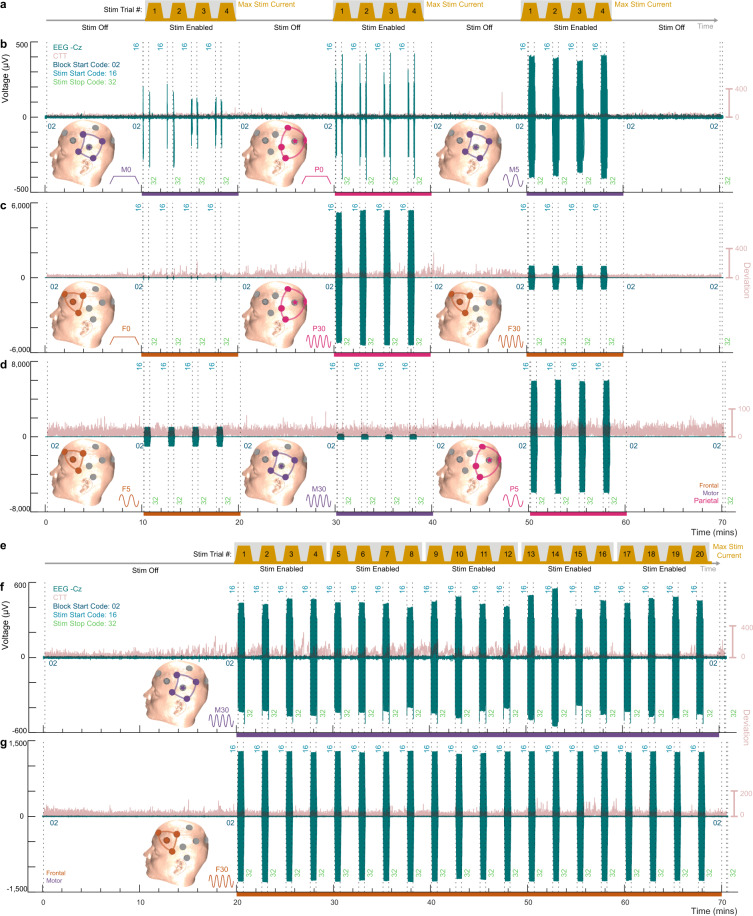
Block implementation with timeseries of a complete experimental series for exemplary participants. (**a**) Session design for Experiment 1. Each session consisted of four 10-min *Stim Off* periods interleaved with three 10-min *Stim Enabled* periods A given stimulation condition was assigned to each *Stim Enabled* period, and applied in 4 consecutive 30 sec trials (Stim Trial # 1, 2, 3, 4). Experimental implementation, including EEG (Cz electrode) and CTT timeseries, for participant 10’s (**b**) first, (**c**) second, and (**d**) third sessions of Experiment 1. (**e**) Session design for Experiment 2. EEG and CTT timeseries for participant 24’s (**f**) first and (**g**) second session implementation. One stimulation condition (either M30 or F30; cartoon insets) was repeatedly applied during each session. In each session of Experiment 2, an initial 20-min *Stim Off* period, was followed by 50-min *Stim Enabled* period which included 20 trials of stimulation. Timeseries triggers in EEG data (vertical dashed lines) are marked with trigger codes. Across all sessions, for both experiments, EEG (teal) and CTT performance (pink), as well as physiology (not illustrated), were acquired continuously.

A precise experimental sequence with EEG (channel Cz), behavioral timeseries, and associated triggers are illustrated for Experiment 1 and Experiment 2 (Fig. 3) for exemplary participants 10 and 24, respectively. This demonstrates participant-wise implementation of the block design in Fig. 2e–h from the data collection perspective. In all experiments, participants continuously performed a task, with associated performance, EEG, and physiology recorded.

At their first session of Experiment 1 (Fig. 3b), participant 10 was pseudo-randomly assigned to receive stimulation conditions M0, P0, and M5 (indicated by insets). Each stimulation condition was repeated over 4 consecutive trials in a block (*Stim Enabled)*; for each trial current was ramped up (over 5 secs), sustained for 30 secs at a maximum designated current intensity (0.5 or 1 mA; *Max Stim Current*), then ramped back down. Session 2 of Experiment 1, for participant 10, continued similarly with F0, P30, and F30 stimulation applied during the stimulation enabled blocks (Fig. 3c). Session 3 of Experiment 1, for participant 10, also continued similarly with F5, M30, and P5 stimulation applied during the stimulation enabled blocks (Fig. 3d).

In session 1 of Experiment 2 (Fig. 3f), participant 24 received the M30 stimulation condition (indicated by insets). The M30 stimulation condition was repeated over 20 consecutive trials divided across five consecutive stimulation enabled blocks (*Stim Enabled)*; for each trial current was ramped up (over 5 secs), sustained for 30 secs at a maximum designated current intensity (0.5 or 1 mA; *Max Stim Current*), then ramped back down. Session 2 of Experiment 2, for participant 24, continued similarly with the F30 stimulation condition applied during the stimulation enabled blocks (Fig. 3g).

The band-pass filtering applied in Fig. 3 for display purposes, produces large voltage artifacts in the EEG during ramp-up and ramp-down, as well as removing DC voltage artifacts, which is present in unfiltered EEG-stimulation data^21^.

## Data Records

All raw data described in the text^87^ can be accessed directly at: 10.5281/zenodo.3837212. The main data repository contains raw data for Experiment 1 and 2, in*.cnt* format (see *EEG and Behavioral Data*)^87^; in addition, down sampled data for Experiment 1 and 2 are indexed in linked repositories^88,89^ in*.mat* format, which is compatible with MATLAB and Python. The dataset is also provided according to the Brain Imaging Data Structure (BIDS)^90^ specifications and can be accessed directly at: 10.18112/openneuro.ds003670.v1.1.0. Trial-wise plots of data are fully indexed for EEG PSDs^91^: 10.6084/m9.figshare.14810517.v1, spectrograms^92^: 10.6084/m9.figshare.14810442.v1, and topoplots^93^: 10.6084/m9.figshare.14810478.

All experimental sessions and applied stimulation conditions are summarized in Table 1 and Table 2 for Experiment 1 and Experiment 2, respectively (see also the *Sessions Summay.xlsx* file in the main data repository). The *Sessions Summay.xlsx* file contains the participant numbers (*Sub#*), participant session label, participants’ session number, stimulation conditions used for each session (*Arms*), date of the session (in month/date/year), and associated file number for each session (*File Num*). The *Sessions Summay.xlsx* file also breaks down which condition out of 9 (F0, M0, P0, F5, M5, P5, F30, M30, P30) were applied for each session, which block the stimulation type was applied in (*Stim Block 1, Stim Block 2, Stim Block 3*), as well as the stimulation amplitude (in mA) used for each session and each block (*Stim Amp Block1, Stim Amp Block 2, Stim Amp Block 3*). For Experiment 2 the montage and stimulation intensity were used across all respective stimulation blocks (Listed under *Stim Block 1* and *Stim Amp Block1*).

### Demographics, PSQI, and Pre Post questionnaires

The *GX_Subject Info & Behavioral Data.xlsx* file contains paginated and tabulated participant-reported information on demographics (*Demographic*), PSQI responses (*PSQI*), and pre- and post-session questionnaires (*All Behavioral*). Data were digitized and hand-imputed from paper records and repeated participants were indicated by notes made on each participant number and color-coding indicated repeated participants with their updated participant numbers.

Within the *Demographic* page of the *GX_Subject Info & Behavioral Data.xlsx* file the columns indicated each participants’ (rows) participant number (*Sub#*), date of data collection (month/date/year), age (in years), gender (Male or Female), height (in cm), weight (in kg), ethnicity, race, years of education, handedness (left or right handed), whether English was their first language, their proficiency in English, whether they exercise or not, how many hours they exercise, if they’ve ever had electrical stimulation before, and how recently have they had electrical stimulation.

Within the *PSQI* page of the *GX_Subject Info & Behavioral Data.xlsx* file the data were arranged by participant (rows) and PSQI questions (columns). Each column other than participant number (*Subj#*) and date, are labeled according to their correspondence to each PSQI question. For example, *PSQI-5-a* corresponds to PSQI question number 5, part a, asking: *During the past month, how often have you had trouble sleeping because you cannot get to sleep within 30 minutes?* Notes were made when participants indicated responses that were outside the scope of the questionnaire or if decisions were made to average or quantize participants’ responses, when appropriate. The full list of PSQI questions as well as scoring the PSQI can be found here^94^.

Within the *All Behavioral* page of the *GX_Subject Info & Behavioral Data.xlsx* file data were arranged according to participant number (*Sub#*), session label, session number, file number (*File Num*), a count of all records, date of data collection, presumed start and end times of each session, stimulation amplitude used for each session and each montage (one number indicates that a single stimulation amplitude was applied for all stimulation montages within a session), session type/montage order (*Arms*), responses to all questions in the Pre-Questionnaire (PQ, numbered by question), pre and post responses to adverse events form (ADpre- for pre stimulation and ADpost- for post stimulation), and all of the pre post rating scales (all called *Karl* to indicate a Karolinska-like scale was administered) including sleepiness (*Karol-Sleep-Pre* or *Post*), discomfort (*Karol-Disc-Pre* or *Post*), pain (*Karol-Pain-Pre* or *Post*), mood (*Karol-Mood-Pre* or *Post*), anxiety (*Karol-Anx-Pre* or *Post*), and energy (*Karol-Energy-Pre* or *Post*). A full list of the questions asked before and after each experimental session can be found in *GX_Demo_PreQuest_Scales.pdf*.

### EEG and Behavioral data

All data records are arranged by experiment (Experiment 1 and Experiment 2). The raw data are sorted by each experimental session. The experimental sessions are named according to the participants’ assigned number and that participant’s experimental session number. For example, for participant 6, session 2 the corresponding folder will be 0602.

Within each session folder is the raw EEG, ECG, and EOG data. These data were exported to*.cnt* format and accompanying*.evt* file, without any applied filters or data augmentation. Each*.cnt* file is labeled with a project pseudonym (*GX*) followed by the participant number, year of data acquisition, month of acquisition, date of acquisition and end time of acquisition in 24-hour clock notation. For example, *GX_07_2019-11-15_20-06-07.cnt* indicates that this data file was collected from participant 7, on November (11) 15^th^, 2019 and the recording ended at 20:06:07 hrs (08:06:07 PM). The session folder also contains a.*mat* file and a.*txt* file. These files contain session timers as well as the montage/s entered for each session. Within the MATLABfilestream < participant number > .mat files relevant variables include *Montages*, and *stim*. For Experiment 1 the *Montages* variable contains the three stimulation conditions that were applied for the defined session. For example, for participant 8 the *Montages* file for session 2 (0802) indicates ‘P30’,’F0’,’P0’. This indicates that the P30 stimulation was applied in stimulation block 1, whereas F0 was applied during stimulation block 2, and P0 was applied during stimulation block 3 (see Fig. 3a*Stim Enabled* time periods). For Experiment 2 the *Montages* variable contains the single stimulation condition that was applied for the whole session, the stimulation condition was repeated three times within the variable for code execution dependencies. For example, for participant 19 the *Montages* file for session 2 (1902) indicates ‘M30’, ‘M30’,’M30’. This indicates that the M30 stimulation condition was applied for all stimulation trials (20 trials total) during session 2 (see Fig. 3e*Stim Enabled* time periods). The *stim* variable within the MATLABfilestream < participant number > .mat files on the other hand kept track of parameters that were passed to PEBL and variables defined within MATLAB for file execution. The *amp* variable defined the stimulation amplitude that was intended to be applied during each session (see Table 1, 2 for stimulation amplitudes applied). The *blocklenmins* and the *blocklen* variables defined the length of each block as outlined in Fig. 2e,g in units of mins and secs, respectively. The variables *timeon*, *rampup*, *rampdown*, and *repeat* defined the length of time stimulation was applied for, the ramp up and ramp down times, and the amount of times stimulation was repeated within a block (Fig. 2f,h), respectively. Other variables within the *stim variable* and within the MATLABfilestream < participant number > .mat files contained parameters that tracked parallel experimental timelines. The text files, MATLABfilestream < participant number > .txt were used as additional time parameter trackers and were not pertinent to any study outcome. Within each session folder is also a folder with identical session naming. This subfolder contains the exported CTT data as well as a CTT summary in*.csv* and*.txt* formats, respectively. The.*cnt* and.*evt* can be used in both MATLAB and python using free and open access accompanying libraries^95^.

The CTT data are located within each session’s folder in a subfolder with an identical name. For example, for participant 19 session 2, their CTT data can be found by navigating through *1902 > 1902 > ptracker-1902.csv*. The ptracker- < participant number + session number > .csv file contains metrics collected from the trackball. Some of these useful metrics include the participant number (*subnum*), the time stamp (time), the cursor’s x-position (*posX*), the cursor’s y-position (*posY*), the change in time steps in ms (*timeDelta*), and the cursor’s radial deviation from the center of the screen (*deviation*). For additional details on CTT output see PEBL’s ptracker documentation.

The physiological (EEG, ECG, EOG) and the behavioral (CTT) data can be time aligned by using triggers sent to the EEG amplifier. The start of the CTT is indicated by the first *Block Start* trigger (code:02), which can be found in the EEG data once it has been imported for post processing. The start of each stimulation trial is indicated with a stimulation start trigger (code:16). After the stimulation start trigger was delivered, to the stimulator and EEG amplifier, the stimulation current was ramped up over the course of 5 secs until it reached the desired intensity. After 30 secs of stimulation at the desired intensity a stimulation stop trigger (code:32) was delivered to the stimulator and EEG amplifier, and the stimulation intensity was ramped down over the course of 5 secs. This process repeated 4 times for each stimulation enabled block.

## Technical Validation

Our technical validation consisted of consulting computational current flow modeling, quality control of behavioral metrics, and quality control of EEG and ECG voltage changes.

**Fig. 4:**
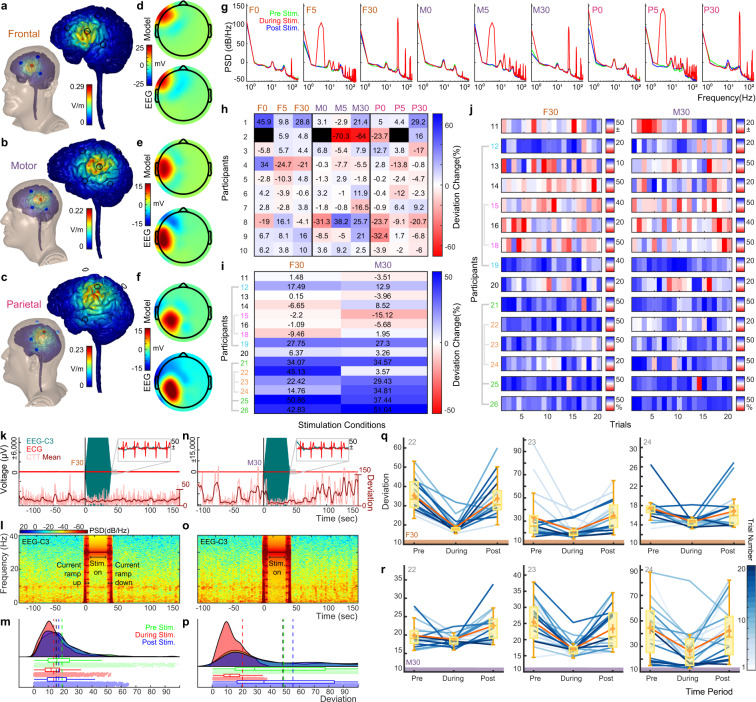
Technical validations. (**a–c**) A high-resolution MRI-derived finite element method (FEM) model computed for frontal, motor, and parietal stimulation montages. These quasi-static models are generalizable to 0, 5, and 30 Hz. (**d–f**) FEM model-predicted voltage topography (*Model*) during stimulation compared with single-participant (participant 10) peak scalp voltage (*EEG*) for frontal, motor, and parietal stimulation. (**g**) Baseline corrected EEG Welch power spectral density (PSD) computed over 30-sec periods pre, during, and post stimulation for participant 10 over all 9 stimulation conditions. (**h**) Percent change in deviation for behavioral (CTT) results for Experiment 1 averaged across 4 trials of each indicated stimulation condition. Data are summarized across participants and stimulation montages. Blacked out boxes indicate missing data. (**i**) Percent change in deviation for behavioral (CTT) results for Experiment 2 averaged across 20 trials of each indicated stimulation montage. Data are summarized across participants and stimulation montages. (**j**) Percent change in deviation for Experiment 2 across participants on a trial level for each of the 20 stimulation trials. Colorbars indicate the minimum (red), maximum (blue), and no change (white) in mean CTT deviation during 30 sec of stimulation compared to 30 secs before stimulation. For panels (**i**) and (**j**) repeated participants are indicated with individual colors and linked gray bars. Exemplary timeseries trials (trial 1) for an (**k**) F30 and (**n**) M30 session. Timeseries indicates combined EEG (channel C3), ECG, and CTT data (raw and moving average) before, during, and after stimulation; with an expanded view (inset) of EEG and ECG data. Spectrogram for an (**l**) F30 stimulation trial, derived from the EEG timeseries trial in panel (**k**) and spectrogram for an (**o**) M30 stimulation trial, derived from the EEG timeseries trial in panel (**n**). One trial of behavioral (CTT) data, for an (**m**) F30 and (**p**) M30 stimulation trial, comparing 30 secs before (*Pre*), during, and after (*Post*) stimulation. Mean deviation is indicated by dashed vertical lines. Panels (**k–p**) all show exemplary data for participant 24. (**q,r**) Behavioral (CTT) deviation from the center of the annulus, for one participant who repeated Experiment 2, three times with F30 and M30 montages. Each experimental run is indicated with a different participant number (1st run = *22*, 2nd run = *23*, 3rd run = *24*). Deviation scores are indicted for 30 secs pre, during, and post stimulation. Mean deviation is indicated in orange, median is indicated in yellow, and each trial is indicted in shades of blue.

A high-resolution finite element model (FEM) was generated based on an MR image (1 mm^3^ voxel size) in order to predict current flow pattern in three different montages used in the study (i.e. frontal, motor, parietal). The head model consisted of seven different tissues layers with conductivities assigned to each: skin (0.465 S/m), skull (0.01 S/m), fat (0.025 S/m), CSF (1.65 S/m), air (10^−15^ S/m), gray matter (0.276 S/m) and white matter (0.126 S/m). The resulting masks were then meshed using ScanFE (Simpleware, LTD, Exeter, UK) and solved in a FEM solver (COMSOL, Burlington, MA, USA). The total stimulating current in each montage (i.e. frontal, motor, parietal) was applied at the center electrode and the four (three for frontal) surrounding electrodes were assigned as ground. The FEM model predicted the expected magnitude of current (electric fields) reaching underlying brain tissue and that electrode montages encapsulated the 3 brain regions of interest (frontal, motor, parietal; Fig. 4a–f). Mean EEG recordings of scalp voltages during stimulation, were in accord with the magnitude and spatial distribution of FEM model predictions of scalp voltage for each montage (Fig. 4d–f). To examine the designated frequency of stimulation applied during each stimulation trial, the Welch power spectral density (PSD; Fig. 4g); during stimulation topoplots; and time-frequency spectrograms (Fig. 4k–p) for EEG data were computed for pre, during, and post stimulation, accordingly, for all participants. Together these metrics aided in corroborating the applied stimulation frequency (with the PSD and spectrograms) as well as the stimulation’s spatial location (with the topoplots). The stimulation voltage artifacts in the trial-wise timeseries data (Fig. 4k,n) also aided in stimulation montage and frequency corroboration. These participant and trial-wise data are fully indexed for EEG PSDs^91^: 10.6084/m9.figshare.14810517.v1, spectrograms^92^: 10.6084/m9.figshare.14810442.v1, and topoplots^93^: 10.6084/m9.figshare.14810478. In addition, we extracted and tabulated the PSD at the most dominant frequency during stimulation for all participants and all trials, across both experiments (see Online-only Table 1 for Experiment 1 and Online-only Table 2 for Experiment 2). All dominant frequencies matched the frequency of programmed stimulation (i.e. 0 Hz for F0, M0, P0; 5 Hz for F5, M5, P5; and 30 Hz for F30, M30, P30). These are tabulated together with behavioral data (CTT deviation change) for each trial of all participants across both experiments. These tabulated data also contain trial-wise means and standard deviations, whereas group-wise sample sizes, means, standard deviations, and confidence intervals can be found in Table 4. Metrics for participants who repeated the experiment were averaged across repeats before being added to group-wise metrics.

**Table 4:** Experiment 1 and 2 group-wise CTT deviation change and EEG PSD.

Experiment	Stim Type	Sample Size	CTT Deviation Change (%)			EEG PSD (dB/Hz)		
			Mean	Std	95% CI (+/−)	Mean	Std	95% CI (+/−)
1	F0	9	8.02	19.98	15.36	146.01	10.66	8.19
	F5	10	0.77	11.72	8.38	145.15	10.53	7.53
	F30	10	4.69	12.93	9.25	149.61	9.90	7.08
	M0	9	−2.94	11.50	8.84	157.31	7.51	5.77
	M5	10	−4.28	26.74	19.13	145.97	7.57	5.42
	M30	10	0.26	26.18	18.73	145.94	7.56	5.41
	P0	10	−6.47	14.78	10.57	160.78	11.46	8.20
	P5	9	−2.56	7.47	5.74	155.26	11.06	8.50
	P30	10	−0.39	14.98	10.72	148.55	9.87	7.06
2	F30	9	9.67	17.17	13.20	152.04	9.15	7.04
	M30	9	8.42	16.38	12.59	155.57	3.50	2.69

The EEG PSD (dB/Hz) at the most dominant frequency during stimulation at electrode FC5 for frontal stimulation, C3 for motor stimulation, and CP5 for parietal stimulation; as well as the CTT deviation change (%) is tabulated across participants and all trials for each stimulation condition. The sample size, mean, standard deviation (*Std*), and 95% confidence interval (*95% CI* (+/−)) for each metric is also tabulated.

CTT data were examined after each experimental session to ensure data were a continuous stream that approximated the time of the EEG, ECG and EOG recordings. Since CTT data were sampled at the frequency of the experimental monitor’s screen refresh rate, data were sometimes nonuniformly sampled at 60 Hz. This nonuniformity was eliminated *post hoc* with uniform sampling using the *resample* function in MATLAB. The CTT data were then examined 30 secs before, during, and after stimulation. The mean across trials for each time period was computed for each stimulation montage across participants for both Experiment 1 and 2 (Fig. 4h,i). For Experiment 2, we examined the average percent change in deviation (taken over 20 trials) for each participant and each stimulation type (F30 and M30; Fig. 4i). We then expanded our examination to look at each participants’ performance (percent change in deviation) on a trial-to-trial level for each stimulation type (F30 and M30; Fig. 4j). Four of the participants who repeated the experiments were denoted with unique participant numbers including participant number 12,19; participant number 15, 18; participant number 21, 25, 26; and participant number 22, 23, 24 (Fig. 4i,j). Two trials for session 1 and 2 for participant 24 are examined in detail from different perspectives including as a timeseries signal plotted with concurrent CTT (Fig. 4k,n); as a time-frequency breakdown (spectrogram) of the EEG voltage from C3 (Fig. 4l,o), and as behavioral comparison of CTT data pre, during, and post stimulation (Fig. 4m,p). Behavioral CTT data for all three repeats for participant number 22, 23, 24 were examined in detail for F30 and M30 stimulation montages (Fig. 4q,r).

During EEG, ECG, and EOG data collection; data were monitored in real time to ensure continuous data streaming. For participant 01 session 01 and session 02, data collection was halted at the end of the sessions (0101 and 0102) due to a technical error. Session 0101 contained 1 trial of F0 for ~2 mins, whereas session 0102 contained 1 trial of F0 for 30 sec. For participant 02 session 01 (data record 0201), the first trial of F30 contained a ramp-up/down time of 30 secs rather than 5 secs. Subsequent trials for 0201 contained a 5 sec ramp-up/down time. EEG amplitudes over time were examined to confirm expected stimulation-generated scalp voltage (voltage artifact see^21,22^) during stimulation and concurrency with ECG and CTT data (Fig. 4k,n). EEG spectra during stimulation were examined to ensure that they contained significant power at the frequencies of stimulation (0 Hz, 5 Hz, 30 Hz; Fig. 4l,o). The CTT deviations, for the aforementioned examinations, were computed and its distributions were examined on a trial-by-trial basis (Fig. 4q,r).

## Data Availability

The latest version of all accompanying code for this dataset can be acquired within this repository: https://github.com/ngebodh/GX_tES_EEG_Physio_Behavior. MATLAB, version 2018b and 2019b were utilized with functions from EEGlab^96^, Raincloud plots toolbox^97^, and ANT neuro’s import functions^95^.

## Online-only Tables

**Online-only Table 1:** Experiment 1 trial-wise CTT deviation change and EEG PSD.

Participant	Stim Type	EEG PSD (dB/Hz) Trials				EEG PSD (dB/Hz)		CTT Deviation Change (%) Trials				CTT Deviation Change (%)	
		1	2	3	4	Mean	Std	1	2	3	4	Mean	Std
**1**	**F0**	144.11				144.11		45.90				45.90	
	**F5**	148.45	147.91	148.09	148.08	148.13	0.23	12.79	1.89	1.87	22.63	9.80	10.00
	**F30**	151.00	150.91	151.57	151.41	151.22	0.32	28.16	19.69	22.74	44.75	28.80	11.20
	**M0**	161.73	161.55	161.28	161.03	161.40	0.31	15.51	7.11	4.87	−14.91	3.10	12.90
	**M5**	141.45	140.94	141.77	141.53	141.42	0.35	−1.38	−7.70	4.20	−6.66	−2.90	5.50
	**M30**	135.13	135.54	134.74	135.59	135.25	0.40	32.00	7.57	19.19	26.74	21.40	10.60
	**P0**	154.00	153.56	153.40	152.89	153.46	0.46	25.39	8.19	−7.40	−6.01	5.00	15.30
	**P5**	149.14	149.16	148.93	149.19	149.11	0.12	4.16	16.58	−0.40	−2.80	4.40	8.60
	**P30**	149.00	148.95	149.10	149.20	149.06	0.11	26.40	26.47	25.89	37.93	29.20	5.80
**2**	**F0**												
	**F5**	147.80	147.96	148.05	147.78	147.90	0.13	4.91	46.83	47.03	−75.16	5.90	57.60
	**F30**	141.06	147.27	147.08	147.36	145.69	3.09	31.62	19.90	−48.15	15.95	4.80	35.90
	**M0**												
	**M5**	151.83	152.73	152.46	151.96	152.25	0.42	−0.24	−232.18	−38.34	−10.59	−70.30	109.10
	**M30**	152.41	152.80	151.92	151.60	152.18	0.53	−111.52	76.66	−63.59	−157.40	−64.00	101.30
	**P0**	142.90	147.60	147.90	146.92	146.33	2.32	−67.98	−28.12	−8.55	9.85	−23.70	33.30
	**P5**												
	**P30**	136.81	135.67	134.01	134.25	135.19	1.31	71.96	27.99	2.13	−38.27	16.00	46.20
**3**	**F0**	141.75	142.40	142.55	142.23	142.23	0.35	19.39	12.02	−4.22	−50.40	−5.80	31.30
	**F5**	149.32	149.20	149.41	149.81	149.44	0.26	0.45	16.75	10.67	−5.10	5.70	9.80
	**F30**	148.84	148.98	149.44	148.57	148.96	0.36	7.23	−29.48	−39.84	79.63	4.40	54.10
	**M0**	148.01	148.32	147.67	147.85	147.96	0.28	1.15	8.05	12.61	5.19	6.80	4.80
	**M5**	142.31	142.73	142.22	143.18	142.61	0.44	−8.75	5.39	−13.79	−4.42	−5.40	8.10
	**M30**	143.34	142.68	143.10	142.65	142.94	0.34	3.46	27.17	21.58	−20.61	7.90	21.50
	**P0**	138.71	138.40	138.96	140.07	139.04	0.73	15.02	−4.79	24.72	16.05	12.70	12.50
	**P5**	130.47	131.66	129.35	131.29	130.69	1.02	7.65	−23.34	14.21	16.69	3.80	18.50
	**P30**	130.01	130.55	131.92	130.15	130.66	0.87	−44.56	−37.07	16.54	−3.08	−17.00	28.80
**4**	**F0**	151.44	151.90	150.58	150.52	151.11	0.67	48.04	49.31	12.16	26.45	34.00	17.90
	**F5**	136.74	136.08	136.32	136.32	136.37	0.27	−22.05	4.44	−32.48	−48.55	−24.70	22.30
	**F30**	148.09	148.25	148.11	148.13	148.15	0.07	−18.89	0.31	6.91	−72.50	−21.00	36.00
	**M0**	162.19	161.81	161.14	161.92	161.77	0.45	6.12	14.87	0.40	−22.56	−0.30	16.00
	**M5**	156.94	156.51	156.98	157.44	156.97	0.38	17.67	−52.35	21.24	−17.48	−7.70	34.50
	**M30**	145.40	144.49	144.97	145.24	145.03	0.40	3.50	−20.33	−10.42	5.18	−5.50	12.10
	**P0**	170.31	170.14	170.45	170.40	170.33	0.14	0.02	15.63	−9.88	5.58	2.80	10.70
	**P5**	162.80	163.02	163.03	162.88	162.93	0.11	−1.78	−4.47	−66.54	17.44	−13.80	36.50
	**P30**	151.55	151.71	152.00	151.85	151.78	0.19	−8.06	−2.07	−6.53	13.38	−0.80	9.80
**5**	**F0**	158.69	159.23	158.82	158.85	158.90	0.23	4.98	−18.35	−2.74	4.79	−2.80	11.00
	**F5**	147.60	147.45	146.97	147.08	147.28	0.30	3.23	−25.33	−7.57	−11.40	−10.30	11.80
	**F30**	159.27	158.79	159.15	159.46	159.17	0.28	3.45	9.63	−5.86	11.98	4.80	8.00
	**M0**	160.53	160.34	160.22	159.94	160.26	0.25	−2.37	4.53	−12.15	4.79	−1.30	8.00
	**M5**	145.18	145.79	146.04	145.79	145.70	0.37	10.26	19.20	1.69	−19.74	2.90	16.70
	**M30**	156.21	156.02	155.83	156.00	156.02	0.16	7.72	−4.85	−5.10	−5.12	−1.80	6.40
	**P0**	175.90	175.80	175.87	176.19	175.94	0.17	7.10	5.77	−6.84	−6.98	−0.20	7.70
	**P5**	169.51	169.40	169.58	169.44	169.48	0.08	−0.76	16.70	−4.51	−20.84	−2.40	15.40
	**P30**	152.36	152.77	152.98	153.07	152.80	0.32	0.63	−0.91	−12.34	−6.01	−4.70	5.90
**6**	**F0**	124.28	128.87	132.35	136.10	130.40	5.04	21.76	−3.28	3.90	−5.55	4.20	12.40
	**F5**	124.43	125.18	125.37	125.31	125.07	0.44	−2.18	−3.42	−24.33	14.25	−3.90	15.80
	**F30**	128.65	129.64	128.71	128.40	128.85	0.54	18.83	−17.04	1.08	−5.45	−0.60	15.00
	**M0**	165.44	165.08	165.54	165.47	165.38	0.21	−5.03	12.73	6.98	−2.00	3.20	8.20
	**M5**	156.34	156.15	156.44	156.53	156.37	0.16	4.00	5.45	−18.77	5.51	−1.00	11.90
	**M30**	156.77	155.96	156.13	156.80	156.42	0.43	18.83	19.13	3.73	6.09	11.90	8.20
	**P0**	169.29	169.78	169.38	169.29	169.44	0.23	−5.26	14.22	2.38	−12.75	−0.40	11.50
	**P5**	163.56	163.34	163.50	163.63	163.51	0.12	−9.55	−13.20	−6.89	−18.20	−12.00	4.90
	**P30**	162.44	163.05	163.03	162.75	162.82	0.29	11.08	−2.89	−12.02	−5.47	−2.30	9.70
**7**	**F0**	150.21	149.72	149.89	150.45	150.07	0.33	7.62	−1.05	−4.37	8.95	2.80	6.50
	**F5**	153.50	153.30	153.21	153.10	153.28	0.17	−8.73	−4.19	0.80	0.79	−2.80	4.60
	**F30**	153.04	152.86	152.45	153.03	152.85	0.28	−0.62	13.73	5.09	−2.99	3.80	7.40
	**M0**	142.97	143.83	143.46	142.62	143.22	0.53	11.92	−3.74	−5.51	−9.93	−1.80	9.50
	**M5**	136.72	135.51	136.10	136.02	136.09	0.50	14.73	−5.62	8.26	−20.48	−0.80	15.60
	**M30**	134.55	133.11	134.13	134.75	134.14	0.73	−49.31	−11.22	3.77	−9.24	−16.50	22.90
	**P0**	160.95	162.01	161.20	160.59	161.19	0.60	−4.80	4.36	7.10	−10.12	−0.90	8.00
	**P5**	156.65	156.94	156.97	156.66	156.81	0.17	12.08	7.31	3.01	3.16	6.40	4.30
	**P30**	147.92	147.78	147.20	148.69	147.90	0.61	12.04	16.46	9.29	−0.96	9.20	7.40
**8**	**F0**	134.73	133.33	134.41	135.27	134.44	0.82	−16.79	−0.35	−77.69	18.67	−19.00	41.70
	**F5**	133.82	133.77	133.93	132.69	133.55	0.58	35.05	25.65	−15.78	19.61	16.10	22.20
	**F30**	140.34	139.99	140.27	139.82	140.11	0.24	22.48	−2.17	−27.13	−9.70	−4.10	20.60
	**M0**	161.04	161.14	161.76	162.35	161.57	0.61	−20.77	−53.52	−0.34	−50.42	−31.30	25.40
	**M5**	144.76	146.13	145.69	145.69	145.57	0.58	24.19	44.44	62.49	21.72	38.20	19.10
	**M30**	145.77	145.35	145.60	146.22	145.74	0.37	61.43	12.33	−16.38	45.60	25.70	34.70
	**P0**	165.35	165.19	165.76	165.88	165.55	0.33	−0.41	−85.84	11.38	−20.09	−23.70	43.40
	**P5**	153.82	153.56	153.70	153.74	153.71	0.11	−37.99	3.51	4.88	−6.68	−9.10	20.00
	**P30**	153.40	153.81	153.32	153.43	153.49	0.22	21.57	−14.37	−25.54	−64.35	−20.70	35.40
**9**	**F0**	141.06	141.91	139.16	139.12	140.31	1.40	18.26	9.78	13.17	−14.59	6.70	14.60
	**F5**	148.72	149.31	149.43	149.19	149.16	0.31	3.55	13.44	3.12	12.43	8.10	5.60
	**F30**	160.05	160.26	160.29	160.82	160.36	0.33	−3.40	31.12	8.56	27.89	16.00	16.30
	**M0**	160.90	161.28	161.34	162.00	161.38	0.46	−4.67	17.24	−20.21	−26.21	−8.50	19.40
	**M5**	134.98	135.52	134.80	135.55	135.21	0.38	−6.59	3.54	4.12	−21.12	−5.00	11.80
	**M30**	144.85	146.72	146.04	146.59	146.05	0.85	32.81	−9.05	31.20	28.98	21.00	20.10
	**P0**	160.86	160.13	160.30	160.90	160.55	0.39	−15.35	17.68	−71.54	−60.57	−32.40	41.30
	**P5**	154.31	154.39	153.80	154.32	154.21	0.27	16.17	−9.30	2.03	−2.23	1.70	10.70
	**P30**	143.92	144.34	143.11	142.45	143.46	0.84	13.14	−9.99	−37.04	6.78	−6.80	22.40
**10**	**F0**	163.30	161.95	162.01	162.80	162.52	0.65	33.44	3.05	−0.60	−11.01	6.20	19.10
	**F5**	161.57	161.41	161.34	160.92	161.31	0.28	7.36	3.15	−2.12	6.67	3.80	4.30
	**F30**	161.27	161.07	160.17	160.39	160.73	0.53	22.25	18.99	0.63	−1.93	10.00	12.40
	**M0**	152.91	152.92	152.78	152.81	152.86	0.07	5.21	−5.51	0.89	13.99	3.60	8.20
	**M5**	147.32	147.50	147.66	147.57	147.51	0.14	0.52	12.40	9.16	14.84	9.20	6.30
	**M30**	145.66	145.94	145.28	145.56	145.61	0.27	4.58	1.87	2.43	1.24	2.50	1.50
	**P0**	165.73	165.84	166.13	166.31	166.00	0.27	2.89	−0.57	−20.40	2.63	−3.90	11.10
	**P5**	156.68	156.93	156.92	157.03	156.89	0.15	10.03	−1.40	−12.77	−3.68	−2.00	9.40
	**P30**	158.42	158.59	157.96	158.35	158.33	0.27	−14.11	−39.92	10.53	19.56	−6.00	26.70

The EEG PSD (dB/Hz) at the most dominant frequency during stimulation at electrode FC5 for frontal stimulation, C3 for motor stimulation, and CP5 for parietal stimulation; as well as the CTT deviation change (%) is tabulated for each participant across all trials for each stimulation condition (9). This is then aggregated with the mean and standard deviations (*Std*) across trials.

**Online-only Table 2:** Experiment 2 trial-wise CTT deviation change and EEG PSD.

Participant	Measure	Stim Type	Trial																					
			1	2	3	4	5	6	7	8	9	10	11	12	13	14	15	16	17	18	19	20	Mean	Std
**11**	**EEG PSD (dB/Hz)**	**F30**	166.52	166.48	166.34	166.51	166.56	166.16	166.51	166.31	166.19	166.23	166.46	166.27	166.84	166.68	166.57	166.41	166.54	166.46	166.38	166.77	166.46	0.18
		**M30**	150.77	150.70	151.20	151.00	150.64	150.88	151.91	150.69	149.89	151.01	150.69	150.48	150.72	150.11	150.52	150.30	150.55	150.17	150.26	150.39	150.64	0.44
	**CTT Deviation Change (%)**	**F30**	−2.29	2.11	24.86	22.10	6.73	−3.54	−45.60	−18.19	6.28	19.82	−2.53	28.66	7.94	−1.58	−27.56	2.65	−6.79	−39.21	45.67	10.01	1.48	22.25
		**M30**	4.62	−35.59	−33.49	−25.58	2.34	−2.10	6.66	14.66	2.93	−11.50	−15.52	−0.67	23.50	14.77	29.32	5.67	−30.19	−28.49	2.13	6.30	−3.51	19.05
**12**	**EEG PSD (dB/Hz)**	**F30**	139.64	139.42	140.01	140.03	139.47	139.57	140.35	139.32	139.91	139.75	139.39	139.87	139.77	139.49	139.40	139.16	139.59	139.74	139.65	139.87	139.67	0.29
		**M30**	152.81	152.65	153.11	153.44	152.79	152.55	152.87	152.76	152.52	153.68	152.60	152.85	152.95	153.06	152.68	152.97	153.10	153.66	152.65	153.21	152.95	0.34
	**CTT Deviation Change (%)**	**F30**	34.81	4.79	13.12	24.22	21.48	21.75	20.82	20.32	12.11	15.45	28.82	22.58	16.65	16.79	32.80	20.33	−11.70	4.76	9.89	20.07	17.49	10.48
		**M30**	52.89	5.57	25.98	−0.05	17.16	2.87	18.41	0.36	28.84	33.59	28.19	25.74	9.11	−17.86	8.92	−19.95	13.03	−3.48	1.56	27.08	12.90	17.82
**13**	**EEG PSD (dB/Hz)**	**F30**	148.98	148.87	148.66	148.63	148.61	148.65	148.43	148.91	148.14	148.07	147.84	147.50	147.98	147.56	147.69	147.38	147.23	147.58	147.93	148.03	148.13	0.55
		**M30**	156.85	156.41	156.46	156.12	156.60	156.82	156.20	156.91	156.40	156.63	156.39	156.16	156.10	156.36	156.35	156.42	156.36	156.13	156.10	156.55	156.42	0.25
	**CTT Deviation Change (%)**	**F30**	−14.62	19.16	7.30	17.95	4.84	−5.70	−12.64	−2.01	−32.32	−6.32	4.09	13.72	−4.75	−1.86	−1.21	19.14	−20.16	2.75	6.61	9.02	0.15	13.37
		**M30**	17.09	8.17	−5.72	4.24	−8.79	−5.67	−28.10	6.40	−38.54	−9.78	−2.36	1.93	3.10	17.88	41.56	−82.17	4.89	4.75	6.34	−14.44	−3.96	24.85
**14**	**EEG PSD (dB/Hz)**	**F30**	167.89	167.74	167.79	167.84	167.84	167.83	167.82	167.87	167.73	167.80	167.95	167.90	167.92	167.80	167.71	167.82	167.92	167.97	167.86	168.06	167.85	0.09
		**M30**	159.34	159.46	159.24	159.47	159.59	159.33	159.53	159.47	159.44	159.40	159.79	159.87	159.29	159.53	159.60	159.44	159.40	159.55	159.40	159.50	159.48	0.15
	**CTT Deviation Change (%)**	**F30**	2.77	−8.15	−0.32	−4.93	−11.05	6.85	8.24	2.89	−2.23	−45.99	−0.66	−11.93	−6.36	15.37	−24.43	9.73	−0.75	−4.11	−51.05	−6.86	−6.65	16.80
		**M30**	23.46	−9.67	−16.48	29.78	48.79	8.87	40.64	−1.58	19.88	1.96	18.09	−1.68	24.04	10.50	18.53	−22.70	−30.78	−14.38	3.98	19.09	8.52	20.87
**15**	**EEG PSD (dB/Hz)**	**F30**	157.32	157.46	157.82	158.11	158.08	157.38	157.85	157.92	157.62	158.14	157.94	157.99	157.94	158.31	157.96	157.78	158.17	157.98	157.86	158.05	157.88	0.26
		**M30**	150.24	150.55	150.58	149.99	150.50	150.52	150.43	150.42	150.57	150.42	150.14	150.62	150.54	150.51	150.68	150.23	150.12	150.05	150.52	150.42	150.40	0.20
	**CTT Deviation Change (%)**	**F30**	−5.19	17.25	−8.19	33.30	−5.43	−16.09	5.14	−26.72	−12.44	−4.52	−39.41	1.21	18.94	−1.07	12.42	−25.15	−7.52	22.63	0.28	−3.50	−2.20	17.61
		**M30**	−34.09	−4.51	−4.81	−10.48	−5.35	−5.41	−45.01	−8.33	11.10	−49.36	16.72	−56.84	44.63	1.70	−47.49	4.55	−86.24	−28.08	−4.81	9.80	−15.12	30.42
**16**	**EEG PSD (dB/Hz)**	**F30**	142.48	142.05	142.37	142.22	142.02	142.28	141.89	141.85	141.68	142.30	142.06	142.48	141.99	143.14	141.83	142.76	142.05	142.48	142.13	142.61	142.23	0.36
		**M30**	158.34	158.81	158.70	158.44	158.54	158.46	158.25	158.91	159.04	158.38	158.64	158.82	158.52	158.56	158.78	158.62	158.51	158.80	158.53	158.03	158.58	0.24
	**CTT Deviation Change (%)**	**F30**	−15.55	−3.59	−8.82	−3.57	−16.74	3.23	12.41	0.83	0.00	5.18	3.92	17.57	1.28	−5.26	9.49	−19.06	−14.46	−7.01	6.47	11.91	−1.09	10.37
		**M30**	−11.28	−42.61	5.96	−1.30	24.07	28.13	−25.06	20.65	−25.71	−9.22	6.50	−19.35	−1.22	1.29	2.60	12.32	−19.90	−33.90	−17.98	−7.61	−5.68	19.21
**18**	**EEG PSD (dB/Hz)**	**F30**	144.22	144.17	144.59	144.14	144.09	144.08	144.36	144.40	144.79	144.24	144.30	143.97	144.26	144.22	143.95	144.24	144.21	144.33	144.17	144.28	144.25	0.19
		**M30**	147.21	147.39	148.01	148.02	148.19	148.31	148.86	149.06	149.60	150.82	151.21	151.41	151.64	152.04	152.21	152.40	153.15	153.40	153.01	153.36	150.47	2.18
	**CTT Deviation Change (%)**	**F30**	−28.49	−7.56	−7.00	−70.67	4.96	−14.39	−9.14	−21.50	26.20	26.86	−1.70	−17.51	−23.27	−44.10	22.46	1.83	−23.18	−6.50	1.57	1.91	−9.46	23.17
		**M30**	−25.63	4.77	16.40	6.59	−11.11	39.13	20.17	−18.15	−7.32	−2.18	11.92	36.53	−4.30	8.91	12.91	−9.53	9.22	−10.46	−15.20	−23.74	1.95	18.04
**19**	**EEG PSD (dB/Hz)**	**F30**	147.58	147.41	147.67	147.75	147.33	147.59	147.83	147.64	147.89	147.81	147.84	147.88	147.96	147.85	147.92	147.67	147.80	147.48	148.06	148.01	147.75	0.20
		**M30**	157.11	157.29	157.78	157.69	158.28	157.65	157.46	157.58	157.97	157.66	157.71	157.80	157.38	157.52	157.55	157.86	158.16	157.55	157.86	157.69	157.68	0.28
	**CTT Deviation Change (%)**	**F30**	42.15	38.92	41.99	35.03	34.75	23.75	35.50	45.10	49.24	39.04	13.93	4.10	16.90	20.31	42.33	43.88	−8.72	6.88	14.12	15.79	27.75	16.37
		**M30**	26.76	25.13	22.97	26.63	33.71	35.88	27.10	41.54	39.17	35.21	32.34	40.76	19.72	−3.24	44.29	22.12	25.39	17.20	17.47	15.75	27.30	11.22
**20**	**EEG PSD (dB/Hz)**	**F30**	147.64	148.24	147.92	146.90	147.73	147.27	147.40	147.29	146.96	147.78	147.96	148.56	148.04	148.06	147.94	148.34	147.62	147.88	148.17	148.19	147.79	0.45
		**M30**	154.20	154.15	153.76	153.87	153.91	153.94	153.71	154.17	154.33	153.56	153.22	153.83	154.16	154.13	154.25	154.01	153.96	154.05	153.60	153.63	153.92	0.28
	**CTT Deviation Change (%)**	**F30**	39.30	21.85	5.28	5.98	22.07	10.42	1.58	1.90	26.68	−24.90	2.83	3.05	−21.54	−20.14	25.01	4.55	36.60	−4.98	−25.64	17.52	6.37	19.29
		**M30**	27.84	12.15	1.36	−0.34	−3.47	43.42	22.76	−69.54	−6.17	1.39	6.05	−13.90	28.03	22.84	−7.76	−27.99	12.31	−7.85	35.46	−11.32	3.26	25.11
**21**	**EEG PSD (dB/Hz)**	**F30**	149.16	149.14	150.04	149.44	149.75	149.51	150.03	150.51	150.11	149.72	150.12	150.06	150.23	150.67	150.50	150.10	150.05	149.91	149.87	149.96	149.94	0.41
		**M30**	157.43	157.38	157.89	157.59	157.52	157.44	157.52	157.39	157.29	157.39	157.62	157.37	157.57	157.58	157.53	157.63	157.49	158.20	157.57	157.66	157.55	0.20
	**CTT Deviation Change (%)**	**F30**	59.21	60.43	70.51	16.21	56.77	54.94	45.02	26.17	38.19	29.98	10.54	39.85	53.26	29.75	29.74	54.01	−38.64	4.45	21.70	19.36	34.07	25.20
		**M30**	46.79	47.02	34.99	25.47	37.43	40.63	49.12	55.59	43.98	18.48	8.97	2.80	37.48	23.59	62.22	39.21	53.64	18.05	23.61	22.32	34.57	16.09
**22**	**EEG PSD (dB/Hz)**	**F30**	152.54	152.26	152.52	152.84	152.62	152.99	152.40	152.35	153.04	152.92	152.68	152.72	152.71	152.62	152.20	152.62	152.32	152.46	152.51	153.15	152.62	0.26
		**M30**	161.91	161.63	161.68	162.18	162.01	161.72	161.79	161.90	161.71	161.64	161.98	161.70	161.83	162.21	162.01	161.67	161.74	161.88	161.83	161.67	161.83	0.17
	**CTT Deviation Change (%)**	**F30**	22.42	47.57	44.80	44.34	37.84	34.44	62.98	35.53	46.24	51.19	55.92	26.50	57.18	51.92	44.19	60.46	47.58	39.77	37.77	53.88	45.13	10.75
		**M30**	3.61	−5.49	−5.59	−3.58	−7.49	13.55	24.16	8.10	21.43	−10.44	2.80	−8.70	−3.68	4.85	−16.71	42.91	−9.01	5.38	7.84	7.36	3.57	14.06
**23**	**EEG PSD (dB/Hz)**	**F30**	152.31	151.80	152.04	151.88	151.98	151.81	151.91	152.14	151.80	151.71	151.67	151.83	151.81	151.53	151.60	151.61	151.49	151.42	151.29	151.33	151.75	0.26
		**M30**	156.88	156.88	157.14	156.99	156.90	156.70	157.15	156.51	157.11	157.40	156.66	156.97	156.83	156.86	156.94	157.08	157.54	156.88	156.90	157.47	156.99	0.26
	**CTT Deviation Change (%)**	**F30**	59.54	−13.88	46.48	75.40	65.33	27.31	59.28	24.73	1.93	26.25	22.74	1.73	43.50	27.48	16.67	−34.56	−16.26	1.76	−22.64	35.58	22.42	30.82
		**M30**	34.48	13.02	−10.78	50.66	33.09	19.17	54.57	18.03	22.31	20.51	12.36	44.37	56.32	35.11	33.17	33.28	43.17	23.69	38.09	13.88	29.43	16.56
**24**	**EEG PSD (dB/Hz)**	**F30**	153.62	153.61	153.61	153.77	153.69	153.44	153.76	154.08	153.61	153.26	153.38	153.10	153.27	153.39	153.38	153.48	153.37	153.49	153.12	153.56	153.50	0.23
		**M30**	161.61	161.74	161.43	161.33	161.43	160.94	161.12	161.40	161.45	161.09	161.09	161.41	160.77	160.73	161.07	160.97	161.07	160.98	160.87	160.74	161.16	0.30
	**CTT Deviation Change (%)**	**F30**	34.69	24.79	−8.41	21.03	19.38	17.88	16.84	12.29	18.77	3.25	−3.52	9.02	24.14	23.15	5.70	12.17	−2.67	44.74	7.47	14.57	14.76	12.84
		**M30**	76.37	76.13	49.59	30.41	−25.96	53.52	14.21	48.63	7.73	39.33	68.23	14.54	52.17	36.59	20.90	33.68	−0.25	23.75	15.66	60.99	34.81	26.67
**25**	**EEG PSD (dB/Hz)**	**F30**	151.49	151.71	151.50	151.85	151.45	151.50	151.51	151.48	151.49	151.50	151.58	151.64	151.35	151.54	151.48	152.00	151.86	151.52	151.63	151.92	151.60	0.18
		**M30**	151.80	152.46	152.46	152.92	152.71	152.59	152.64	152.42	152.55	152.90	152.61	152.77	152.76	153.19	152.53	152.86	152.61	152.97	153.09	152.59	152.67	0.30
	**CTT Deviation Change (%)**	**F30**	73.05	54.54	65.04	63.39	53.84	47.41	42.27	67.26	72.11	21.48	57.53	40.54	63.85	−30.94	64.24	63.00	37.96	71.24	28.36	61.03	50.86	24.14
		**M30**	54.27	25.16	43.30	38.44	24.63	46.27	40.34	67.56	33.92	57.42	56.76	53.92	16.34	−26.74	30.28	23.49	30.29	28.61	62.71	41.81	37.44	20.88
**26**	**EEG PSD (dB/Hz)**	**F30**	143.61	143.79	143.47	144.00	144.44	143.89	144.17	144.32	143.96	143.04	143.14	143.13	143.80	144.40	144.58	143.99	144.75	145.12	145.06	144.15	144.04	0.59
		**M30**	155.64	155.58	155.73	155.40	155.48	155.64	155.73	155.77	154.96	155.81	155.65	155.92	155.83	155.69	155.74	156.21	155.82	155.84	156.10	156.05	155.73	0.27
	**CTT Deviation Change (%)**	**F30**	41.15	30.09	42.91	8.14	27.97	30.96	48.50	55.93	26.97	42.86	59.68	36.09	59.05	71.48	64.33	84.88	−5.34	43.52	31.44	55.91	42.83	21.09
		**M30**	71.92	76.84	52.46	58.12	34.41	42.80	60.86	50.02	71.32	−8.93	62.36	35.40	48.39	50.15	50.16	61.83	38.39	52.54	54.93	56.81	51.04	18.23

The EEG PSD (dB/Hz) at the most dominant frequency during stimulation at electrode FC5 for frontal stimulation, C3 for motor stimulation; as well as the CTT deviation change (%) is tabulated for each participant across all trials for each stimulation condition (2). This is then aggregated with the mean and standard deviations (*Std*) across trials for each metric.
